# The surface oxidation effect on photocurrent in WSe_1.95_Te_0.05_ nanosheets

**DOI:** 10.1016/j.isci.2024.111461

**Published:** 2024-11-22

**Authors:** Shiu-Ming Huang, Tzu-Yueh Tu, Pin-Cing Wang, Mitch Chou, Chang-Yu Li, Hao-Ting Wu, Yue-Cheng Hsieh, Ruei-San Chen

**Affiliations:** 1 Department of Physics, National Sun Yat-Sen University, Kaohsiung 80424, Taiwan; 2 Center of Crystal Research, Academy of Innovative Semiconductor and Sustainable Manufacturing, National Cheng-Kung University, Tainan 70101, Taiwan; 3 Department of Materials and Optoelectronic Science, National Sun Yat-Sen University, Kaohsiung 80424, Taiwan; 4 Graduate Institute of Applied Science and Technology, National Taiwan University of Science and Technology, Taipei 10607, Taiwan

**Keywords:** Chemistry, Physics, Materials science

## Abstract

Surface oxidation effect on photocurrent responsibility was detected in WSe_1.95_Te_0.05_ nanosheets, and the photocurrent response depends on the light wavelength. It is enhanced at the wavelength of 405 nm, while showing no change at the wavelength of 532 nm and suppressed at the wavelength of 808 nm. The incident photon-to-current efficiency (IPCE) is expected to increase at 405 nm wavelength, remain unchanged at 532 nm wavelength, and decrease at 808 nm wavelength. Therefore, WO_3_ contributes to the intrinsic properties. The trend of photocurrent change after half-year exposure corresponds to the absorbance change from pristine WSe_1.95_Te_0.05_ to WO_3_. The wavelength-dependent photocurrent responsibility is understood as the wavelength-dependent IPCE of WO_3_ that is from the surface-oxidized WSe_1.95_Te_0.05_.

## Highlights

• Surface oxidation enhances photocurrent response in WSe_1.95_Te_0.05_ nanosheets
• Photocurrent peaks at 405 nm, stays unchanged at 532 nm, and drops at 808 nm
• IPCE expected to increase at 405 nm, consistent with WO_3_ contribution
• Half-year exposure shifts photocurrent, mirroring WO_3_ absorbance trends

## Graphical abstract

**Figure:**
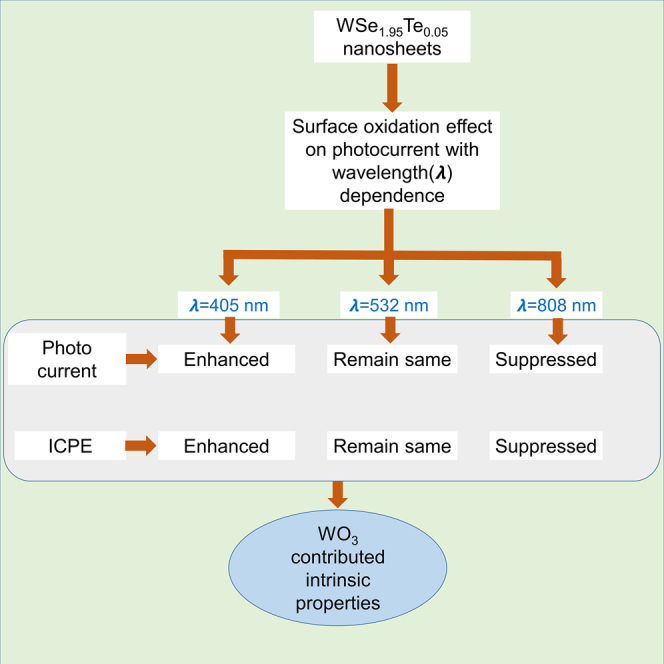

## Introduction

The ideal photodetector is expected to have high photocurrent, fast reaction time, high sensitivity, high efficiency, and ambient condition stability.^1^^,^^2^^,^^3^^,^^4^^,^^5^^,^^6^ The transition metal dichalcogenides (TMDs) have several advantages as candidates for photosensor due to their high photoresponsivity, broadband characteristic, and band-gap tunability.^7^^,^^8^^,^^9^^,^^10^ Moreover, the TMDs have promising minimal defect due to the absence of dangling bond on the surface, which ensures a fast response time without complex mechanics.^11^ The thickness-dependent band gap of TMDs is an intrinsic property that affects the photodetector’s quality.^12^ It is reported that the TMDs would be oxidative in the ambient environment, which severely affects the photocurrent responsibility.^13^^,^^14^ However, rare work study the mechanism of surface oxidation on the photocurrent responsibility.

WSe_2_ is one of the TMDs that has high carrier mobility, strong optical absorption, and high photoconversion efficiency. Contrast to the other members in TMDs, like WS_2_, the oxidation properties of WSe_2_ are relatively less studied.^14^^,^^15^^,^^16^^,^^17^ In this work, we study the surface oxidation effect on photon-to-electron properties of WSe_1.95_Te_0.05_ nanosheets. The photon responsibility was measured under different bias voltage and power before and after half-year exposure in an ambient environment. The result shows that the oxidation influence on the photoresponsivity depends on the incident light wavelength, and it might be enhanced, suppressed, or unchanged. The study supports that the wavelength-dependent photoresponsivity is related to the photon-to-current efficiency of WO_3_ on the surface of the WSe_1.95_Te_0.05_.

### Experimental method

Chemical vapor transport is adopted to grow tungsten diselenide doped with tellurium WSe_1.95_Te_0.05_ single crystals. 99.99% tungsten powder, selenium, and tellurium were introduced into a silica ampoule. The sample was then evacuated to a pressure of 10^−3^ torr. The first step is to synthesize the raw materials into poly crystalline powder. The ampoule was slowly heated to 600°C over 95 h. Secondly, the sample was annealed at 1,050°C for 96 h. Finally, the annealed polycrystalline materials were sealed into a 20 cm silica tube. It was then placed in the two-zone furnace, the temperature was raised to 1,020°C, and gradually decreased to 980°C in 170 h. After growth, the crystals were furnace cooled to room temperature. The as-grown crystals were cleaved along the basal plane, using a silvery reflective surface, and then prepared for further experiments.

Raman spectroscopy was performed in the HORIBA, HR800 with wavelength 633 nm and scan step of 0.3 cm^−1^. The length, width, and height of sample 1 are 2.171 microns, 2.136 microns, and 44 nm, respectively. The length, width, and height of sample 2 are 1.490 microns, 1.283 microns, and 46 nm. Energy-dispersive X-ray spectroscopy confirmed that the crystal is WSe_1.95_Te_0.05_. Keithley 4200-SCS was used to measure the conductance of two WSe_1.95_Te_0.05_ nanosheets in a two-probe method. The I^+^ and V^+^ are on the same contact point, while the I^−^ and V^−^ are on the same contact point.

### Results and discussion

**Figure 1:**
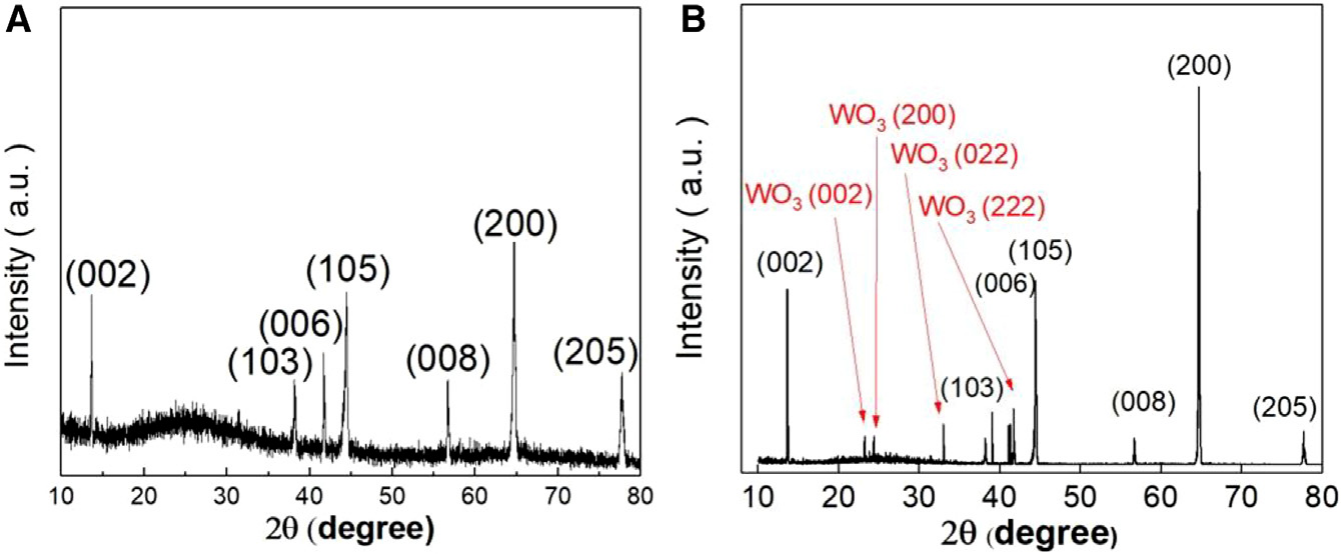
XRD analysis of WSe_1.95_Te_0.05_ (A) The XRD of single crystal WSe_1.95_Te_0.05_. The sharp XRD peaks indicated the highly crystallized structure. The JCPDS card no. of WSe_1.95_Te_0.05_ is #96–9012194 and the PDF card no. is #38–1388. (B) The XRD of single crystal WSe_1.95_Te_0.05_ after oxidation. The WO_3_ JCPDS no. is #83–0950. The new peak at 23°, 24°, 33°, and 43° represents the oxidized WSe_1.95_Te_0.05_-WO_3._

Figure 1 shows the X-ray diffraction (XRD) of the WSe_1.95_Te_0.05_ nanosheets. The sharp peak can be observed, whose full width at half maximum value is 0.2°. The crystal structure was characterized by single-crystalline XRD, which shows a (002) preferable orientation, which is defined as c axis. This sharp peak indicated a good crystalline structure.

**Figure 2:**
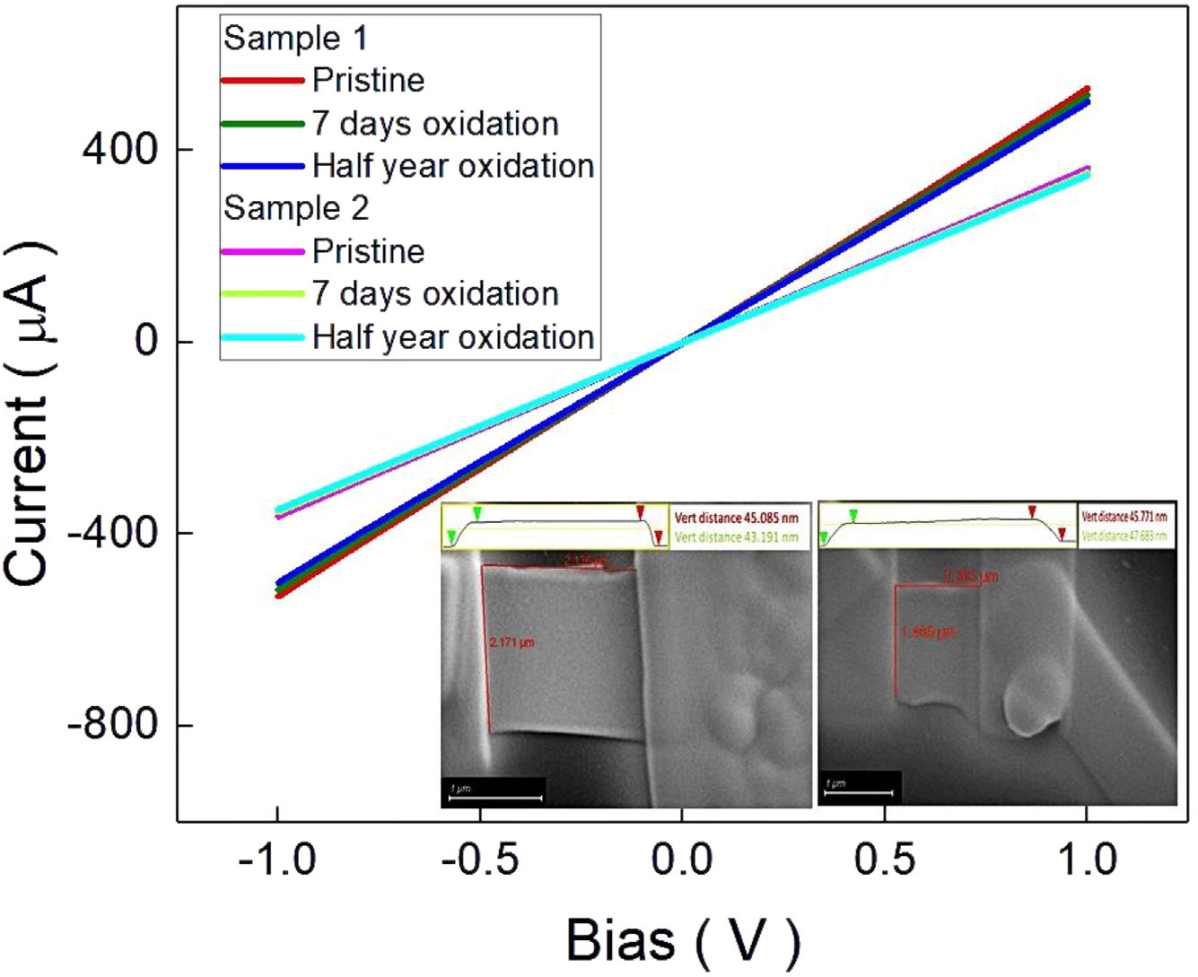
The I-V curves of two samples along with bottom-right inset show the SEM geometric image of sample 1 and sample 2

Figure 2 inset shows the scanning electron microscope (SEM) images of sample 1 (S1) and sample 2 (S2). The SEM image shows that Pt electrodes were deposited on WSe_1.95_Te_0.05_ nanosheets. The length-to-width ratio of S1 and S2 was identical. Atomic force microscopy result shows at the top of inset, and the individual thickness of S1 and S2 was 45 and 46 nm. Figure 2 shows the current-to-bias relation of two samples under pristine and after half-year exposure at ambient environment condition. The half-year exposure under ambient environment does not change the resistivity of the two samples. This implies the air exposure treatment does not change electric transport properties. The detected resistivity is 8.72 × 10^−5^ Ωm (S1) and 1.45 × 10^−4^ Ωm (S2). It is noteworthy that the resistivity is not changed before and after half-year ambient environment exposure. This resistivity difference between two samples might originate from the unavoidable defects induced by the exfoliation process, device fabrication processes, or contribution from the probe wires and electrodes.

**Figure 3:**
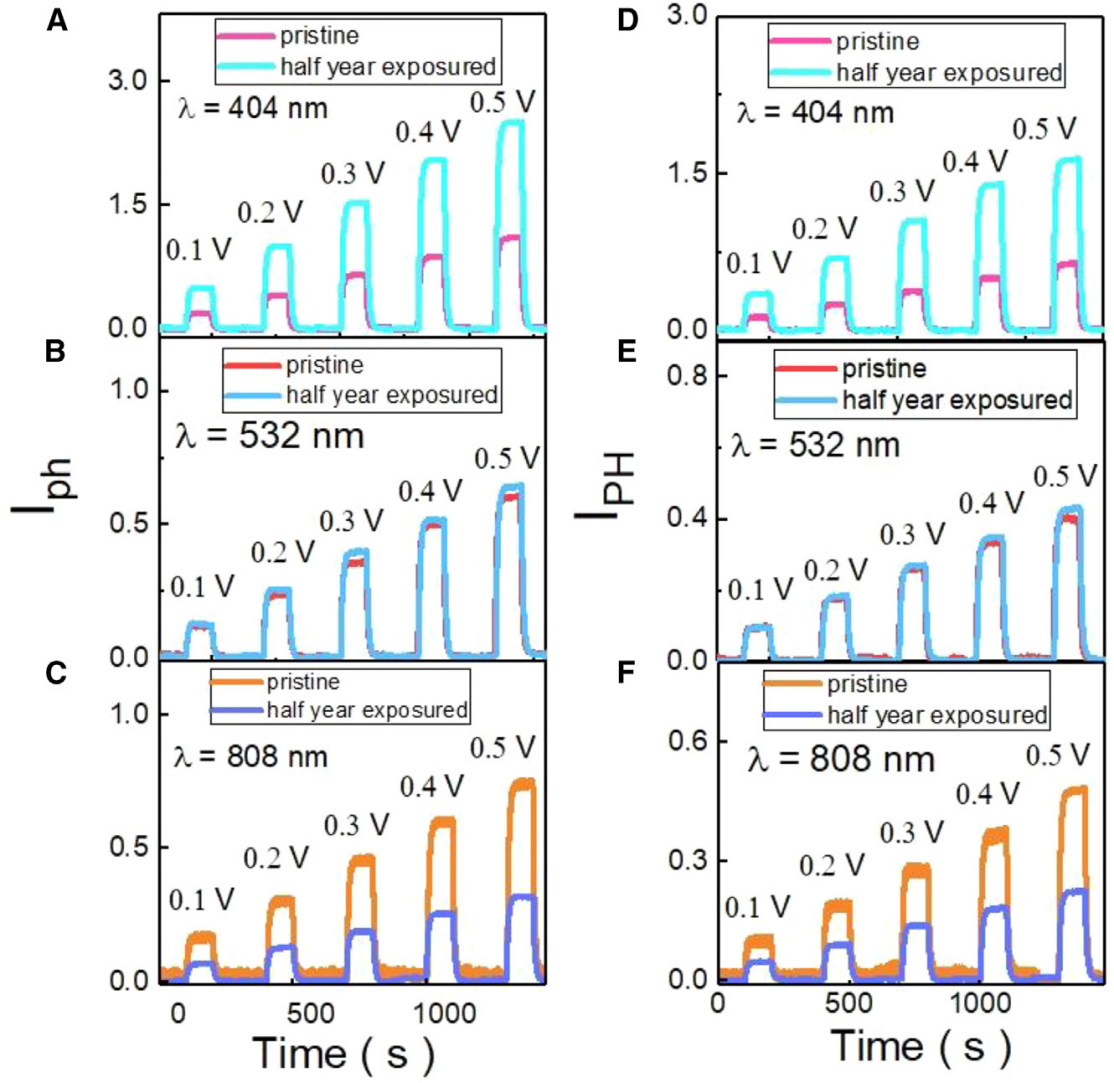
The photocurrent analysis for different applied voltages (A–C) The photocurrent of different applied voltages for sample 1 of as-prepared and after half-year exposure. (D–F) The photocurrent of different applied voltages for sample 2 of as-prepared and after half-year exposure.

Figures 3A–3F show the photocurrent as a function of bias for two samples at the pristine and half-year air exposed. The detected photocurrent is proportional to the bias. The photocurrent of S1 is larger than that of S2 by a factor of 2. This is the same as the ratio of the resistance of two samples. The photocurrent difference might originate from the unavoidable defects induced by fabrication processes. It reveals that the detected photocurrent is enhanced after half-year air exposed at the light wavelength of 405 nm, while the detected photocurrent is the same after half-year air exposed at the light wavelength of 532 nm and detected photocurrent is suppressed after half-year air exposed at the light wavelength of 808 nm. The same characteristic is observed in both samples.

Different from the widely reported observation that surface oxidation severely suppresses the photocurrent in 2D TMDs, our experiment reveals a wavelength-dependent effect.^13^^,^^18^^,^^19^ It is known that resistance is related to the total sample system, while the photocurrent is sensitive to the surface condition due to the short light penetration depth. Our experimental results reveal that the detected resistance does not change, but the photocurrent reveals a wavelength dependence change after the sample exposure to air over half-years. This indicates that the oxidation affects the overall carrier transport characteristic. It is reported that the WO_3_ would form a protected layer on the surface and avoid further oxidation.^20^^,^^21^

**Figure 4:**
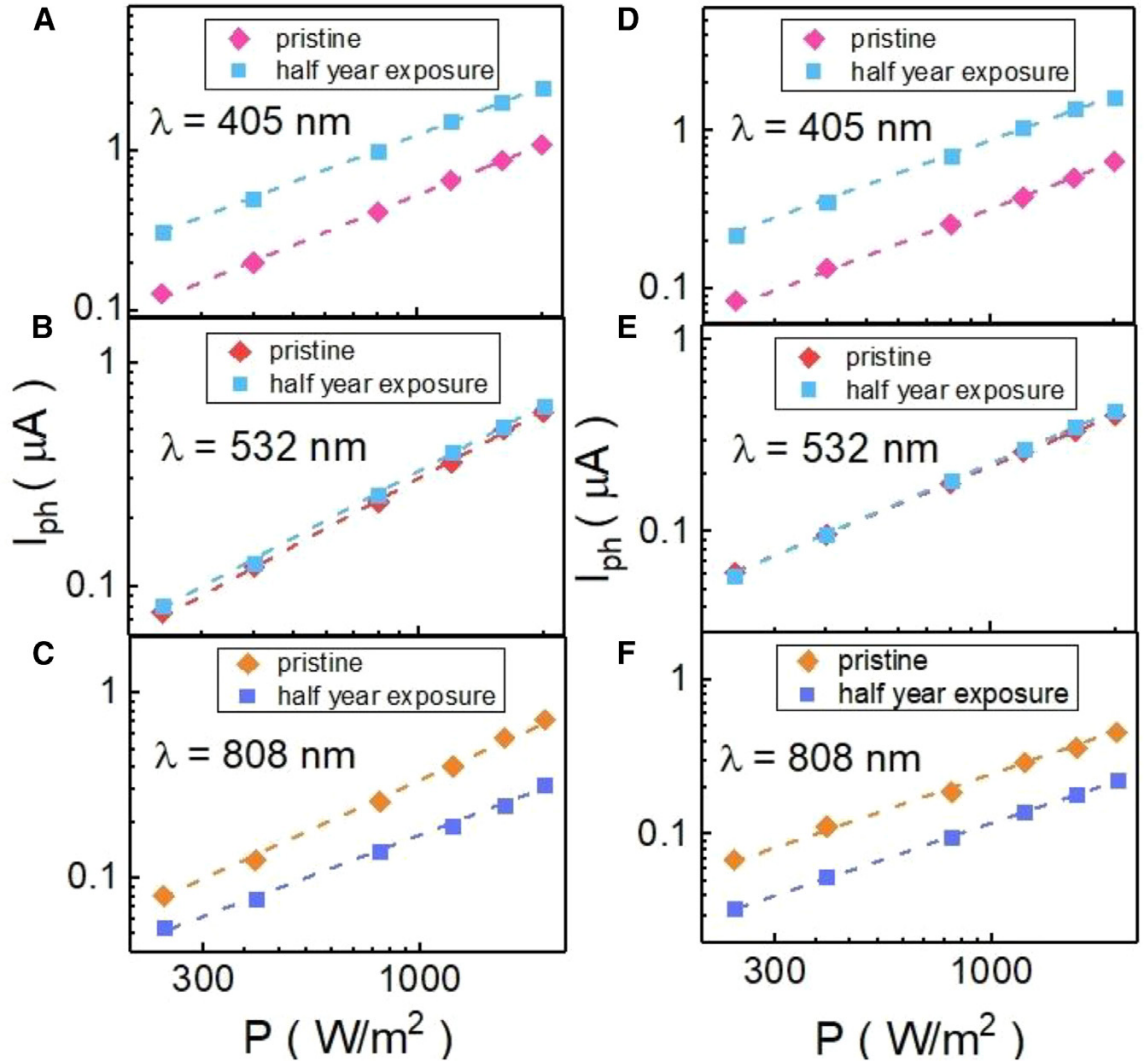
The photocurrent as a function of light power intensity of the two samples (A–C) The photocurrent as a function of light power intensity in pristine and half-year exposed WSe_1.95_Te_0.05_ nanosheets at different wavelengths for sample 1. (D–F) The photocurrent as a function of light power intensity in pristine and half-year exposed WSe_1.95_Te_0.05_ nanosheets at different wavelengths for sample 2.

Figure 4 shows the photocurrent response as a function of light power intensity for pristine and half-year exposure. It reveals that the photocurrent is proportional to the light power intensity in the double-log plot. To identify the relation between the photocurrent and light power intensity, the photocurrent is expressed as *I*_*ph*_ = *AP*^*β*^, where *β* is dependent on the intrinsic carrier transport mechanism. The fitting result is shown in Table 1. The *β* would deviate from the 1 in a system with a complex photon-carrier coupling mechanism. These results indicate that *β* ∼ 1 and that there is no complex photon-carrier coupling mechanism in our system. Furthermore, the *β* is not deviation between pristine and environment exposure, and this indicates that environment exposure does not influence the band structure and the carrier transport property. The photocurrent responsivity is a factor that identifies the photocurrent performance. The photocurrent responsivity (*R*) could be expressed as *R* ∼ *I*_*p*_/*I*, where *I*_*P*_ is the photocurrent and *I* is the light power intensity.

**Table 1:** List of responsivity and *β* of S1 and S2 under different wavelength

Sample	Wavelength (nm)	Responsivity (AW^−1^)	*β*
S1, pristine	405	88	0.98
S1, half-year exposed	405	269	1.02
S1, pristine	532	69	0.98
S1, half-year exposed	532	64	1.00
S1, pristine	808	77	1.04
S1, half-year exposed	808	34	0.89
S2, pristine	405	174	0.96
S2, half-year exposed	405	442	0.95
S2, pristine	532	115	0.93
S2, half-year exposed	532	108	0.89
S2, pristine	808	128	0.88
S2, half-year exposed	808	60	0.94

The applied bias is 0.5 V.

Table 1 lists the extracted the photocurrent and the *β*. The photocurrent current *I_p_*(808nm):*I*_*p*_(532nm):*I_p_*(405nm) ≈ 1:0.89:1.14 for S1 and *I*_*p*_(808nm):*I*_*p*_(532nm):*I*_*p*_(405nm) ≈ 1:0.89:1.35 for two pristine samples. The photocurrent current *I_p_*(808nm):*I_p_*(532nm):*I_p_*(405nm) = 1:2.2:5.2 for S1 and *I_p_*(808nm):*I_p_*(532nm):*I_p_*(405nm) = 1:2.2:5.5 after half-year air exposure. The photocurrent ratio between different light wavelengths is the same for two samples. This indicates that photocurrent change originates from the intrinsic mechanism.

It comes to our attention that the incident photon-to-current efficiency (IPCE), is wavelength dependent for WO_3_.^22^^,^^23^ The *IPCE*(808nm):*IPCE*(532nm):*IPCE*(405nm) ≈ 1:2:5 for WO_3_ and that is close to the observed photocurrent response ratio between different wavelength in our two samples after half-year air exposure (2020Catalysts 10(1) 122).

It is reported that the WSe_2_ would oxidize and form oxidation layer WO_3_, which would protect WSe_2_ to be further oxidized.^24^ As shown in Figure 2, the resistance is the same at the condition of the pristine and after half-year air exposure. That implies that the mechanism of the observed photocurrent change should not be from the entire bulk system. It might only originate from the influence of the WSe_2_ surface condition.

**Figure 5:**
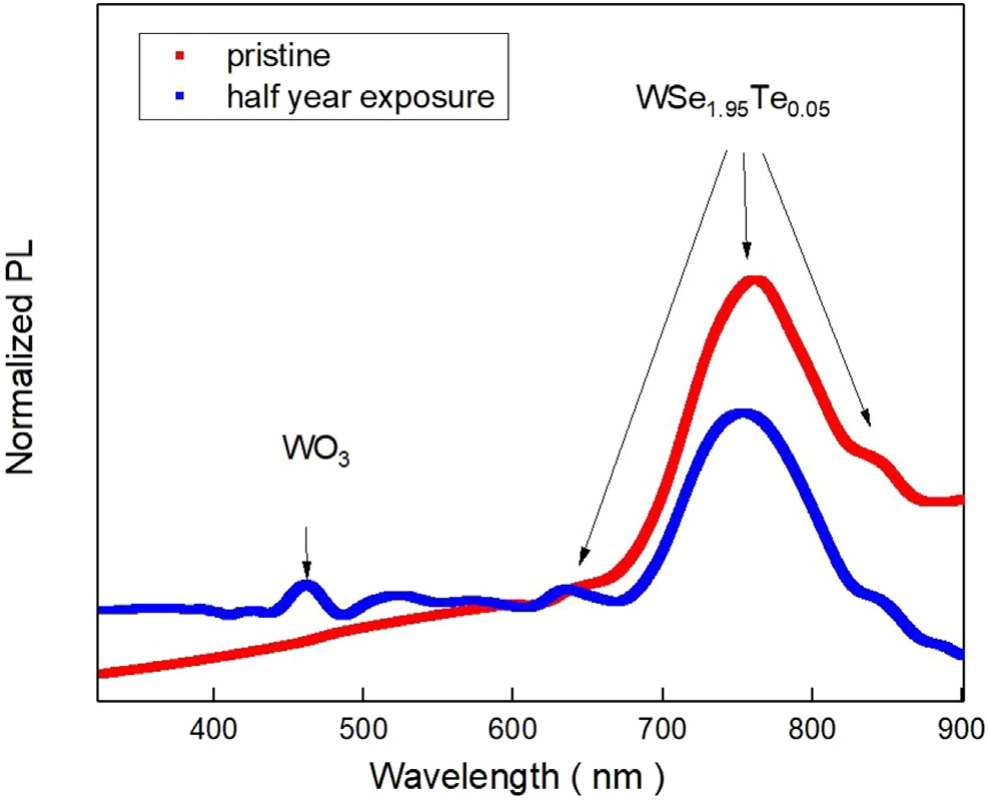
The photoluminescence of WSe_1.95_Te_0.05_

In order to further prove that the increase and decrease in photocurrent is due to the influence of WO_3_ formed by the sample after half-year exposure, the photoluminescence (PL) experiment was conducted with the same sample as shown in Figure 5. Whether the peak near 455 nm in Figure 5 exists, one can distinguish whether the sample is oxidized or not. The peak at wavelength 600 nm is the main peak to identify whether there is WO_3_. In addition, it can also be observed in Figure 5 that the sample after oxidation has a higher signal intensity before the wavelength of 600 nm and a lower signal intensity after 600 nm compared to the WSe_1.95_Te_0.05_. The results in Figure 3 are consistent with the results in Figure 4. Such change of photocurrent of half-year-exposure sample can be taken as the surface oxidation WSe_1.95_Te_0.05_, WO_3_, act as a photon-to-electron transfer layer on the surface of sample. This may significantly affect the IPCE due to the photon incident direction from the surface.^25^

**Figure 6:**
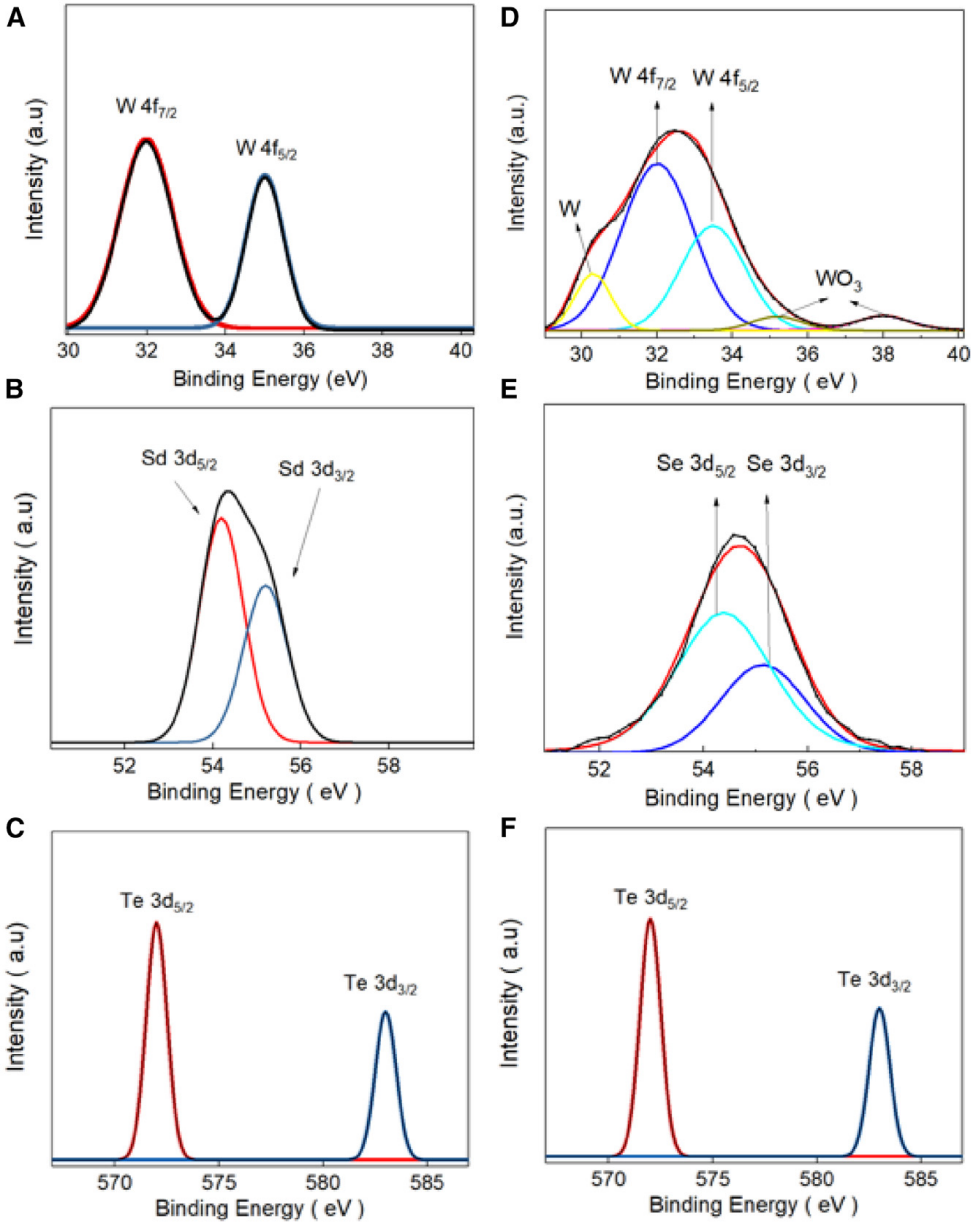
XPS analysis of WSe_1.95_Te_0.05_ (A and D) The XPS spectrum of W4f. The binding energy peak located at 31.9, 35, and 37.4 eV corresponding to the W 4f_7/2_, W 4f_5/2_ of WSe_1.95_Te_0.05_, and WO_3_. (B and E) The XPS spectrum of Se 3d. The binding energy peak located at 54.2 and 55.2 eV corresponding to the Se 3d_5/2_ and Se 3d_3/2_. (C and F) The XPS spectrum of Te 3d. The binding energy peak located at 572.0 and 583.1 eV corresponding to the Te 3d_5/2_ and Te 3d_3/2_.

Figure 6 shows the X-ray photoelectron spectroscopy (XPS) spectrum of the air-exposed WSe_1.95_Te_0.05_ nanosheet. Figures 6A and 6D show the XPS spectrum of W4f. The binding energy peak located at 31.9, 35, and 37.4 eV corresponds to W 4f_7/2_, W 4f_5/2_ of WSe_1.95_Te_0.05_, and WO_3_. Figures 6B and 6E show the XPS spectrum of Se 3d. The binding energy peak located at 54.2 and 55.2 eV corresponds to Se 3d_5/2_ and Se 3d_3/2_. Figures 6C and 6F show the XPS spectrum of Te 3d. The binding energy peak located at 572.0 and 583.1 eV corresponds to Te 3d_5/2_ and Te 3d_3/2_.^26^^,^^27^^,^^28^

**Figure 7:**
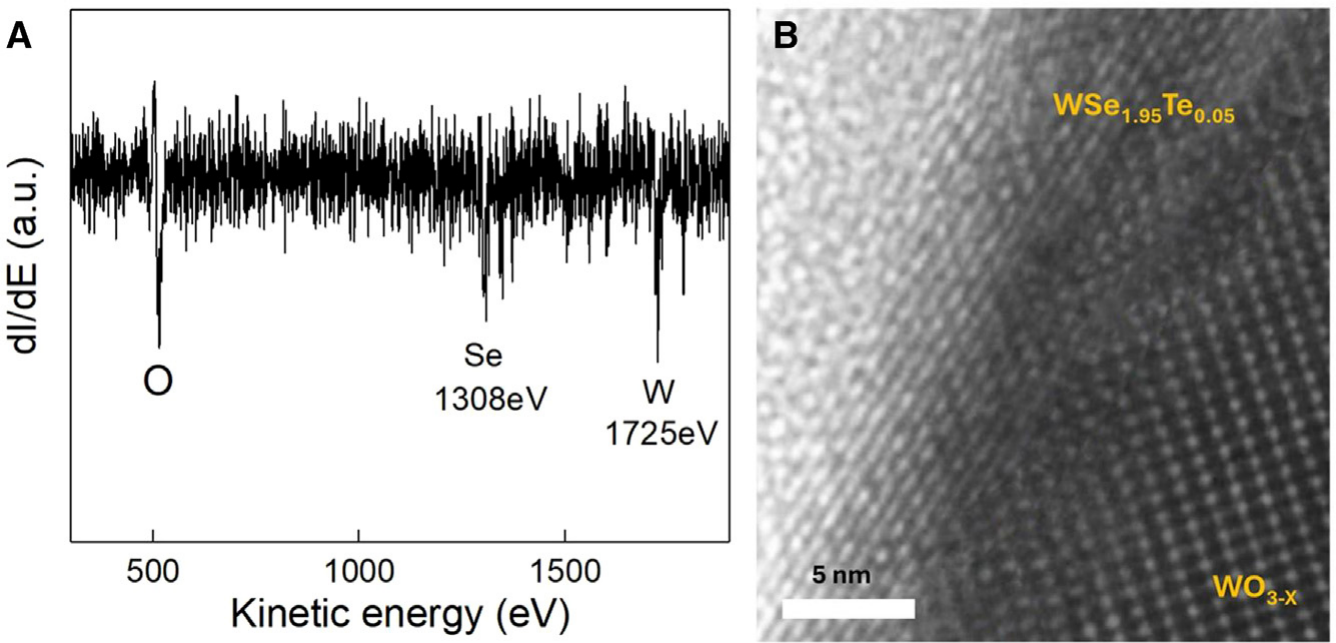
Auger electron spectroscopy and TEM analysis (A) The figure is the result of Auger electron spectroscopy (AES), the peak position of W shifts to lower value from 1,730 eV. This shows the absorption and oxidation on the surface. (B) The TEM images of side view of our sample. The structure of WSe_1.95_Te_0.05_ and WO_3_ is shown in the left side of and lower right corner. The layer structure is the nature properties of van der Waals bonding material.

Figure 7A reveals the Auger electron spectroscopy. Three peaks are related to the Se-LMM, W-MNN, and O-KLL. The peak position at 1,725 eV, which is slightly shifted from the 1,736 eV, indicates the existence of WO_3_ in our nanosheets.^29^ The XPS spectrum supports that no oxidation Se and Te. Figure 7B shows the TEM side view of the half-year-exposure sample. The layer structure and the cluster likely represent the WSe_1.95_Te_0.05_ and WO_3_, respectively. The surface oxidation of WSe_1.95_Te_0.05_ would form WO_3_ on top of the surface and it would form a protective layer to prevent the interlayer structure to be further oxidized. This result also indicated the limited change of photon responsivity by ambient environment exposure.

### Conclusion

Surface oxidation effect on photocurrent responsibility was detected in WSe_1.95_Te_0.05_ nanosheets, and the photocurrent response depends on the light wavelength. It is enhanced at the wavelength of 405 nm, while showing no change at the wavelength of 532 nm and suppressed at the wave length of 808 nm. The IPCE is expected to increase at 405 nm wavelength, remain unchanged at 532 nm wavelength, and decrease at 808 nm wavelength. Therefore, WO_3_ contributes to the intrinsic properties. The trend of photocurrent change after half-year exposure corresponds to the absorbance change from pristine WSe_1.95_Te_0.05_ to WO_3_. The wavelength-dependent photocurrent responsibility is understood as the wavelength-dependent IPCE of WO_3_ that is from the surface-oxidized WSe_1.95_Te_0.05_.

## Resource availability

### Lead contact

For further information and requests related to this study, please contact Shiu-Ming Huang at smhuang@mail.nsysu.edu.tw.

### Materials availability

The materials used in this study, including the WSe_1.95_Te_0.05_ nanosheets, are available upon reasonable request from the lead contact. Specific details regarding the synthesis and preparation of these nanosheets are provided in the Methods section.

### Data and code availability

The photocurrent response data, IPCE calculations, and any analysis code generated during this study are available upon request. The datasets supporting the findings of this study are not publicly available but can be provided by the lead contact on reasonable request.

## Acknowledgments

This work was supported by the 10.13039/501100004663Ministry of Science and Technology, Taiwan through grant no. MOST 111-2112-M-110-012, MOST113-2112-M-110-018, and Center of Crystal Research at National Sun Yat-Sen University Service plan of core-facility center at NSYSU through MOST 110-2731-M-110-001,MOST110-2731-M-110-001, and MOST108 2731-M-110-001.

## Author contributions

S.-M.H.: conceptualization, methodology, and software; T.-Y.T.: data curation and writing – original draft; P.-C.W.: visualization, investigation, and writing – original draft; C.-Y.L.: crystal growth; M.C.: supervision; H.-T.W.: data curation; Y.-C.H.: data curation; R.-S.C.: supervision.

## Declaration of interests

The authors declare no competing interests.

## STAR★Methods

### Key resources table

**Table:** 

REAGENT or RESOURCE	SOURCE	IDENTIFIER
**Chemicals**		

W	Table International Trading Co. Ltd	CAS 7440-33-7
Se	Table International Trading Co. Ltd	CAS 7782-49-2
Te	Table International Trading Co. Ltd	CAS 13494-80-9

### Experimental model and study participant details

The material under study is WSe_1.95_Te_0.05_ nanosheets, which were observed for surface oxidation effects on photocurrent response. The study did not involve biological or human models but focused on nanosheet material properties over a period of six months under controlled environmental exposure conditions.

### Method details

#### Synthesis and preparation of nanosheets

WSe_1.95_Te_0.05_ nanosheets were synthesized and exposed to environmental conditions to promote surface oxidation. Over the course of six months, the material’s structural evolution was studied, focusing on the formation of WO_3_ due to oxidation.

#### Photocurrent measurements

Photocurrent responsibility was analyzed at three different light wavelengths: 405 nm, 532 nm, and 808 nm, using a dedicated photodetector setup. The wavelength-specific response was recorded before and after surface oxidation occurred.

#### Incident photon-to-current efficiency (IPCE) evaluation

The IPCE was calculated at the same wavelengths. Measurements were compared across different stages of surface oxidation to determine how the changes in surface composition (transition from pristine WSe1.95Te0.05 to WO3) influenced the photocurrent.

### Quantification and Statistical analysis

Statistical analysis was performed on photocurrent data to quantify the changes observed at each wavelength. Specifically, enhancements in photocurrent were noted at 405 nm, no change at 532 nm, and suppression at 808 nm. Absorbance data were analyzed to correlate these trends with surface oxidation levels.

### Additional resources

This study did not generate new datasets or additional resources outside of the main methods and analyses. Any additional data or code used for analysis are available upon request from the corresponding author.
